# Associations between *Mycobacterium avium* subsp. *paratuberculosis* antibodies in bulk tank milk, season of sampling and protocols for managing infected cows

**DOI:** 10.1186/1746-6148-9-234

**Published:** 2013-11-27

**Authors:** Casey L Cazer, Rebecca M Mitchell, Kellie M Cicconi-Hogan, Michael Gamroth, Roxann M Richert, Pamela L Ruegg, Ynte H Schukken

**Affiliations:** 1 College of Veterinary Medicine, Cornell University, Ithaca, NY 14853, USA; 2 Department of Population Medicine and Diagnostic Sciences, Cornell University College of Veterinary Medicine, Animal Health Diagnostic Center, Ithaca, NY 14853, USA; 3 Department of Animal and Range Sciences, Oregon State University, Corvallis, OR 97331, USA; 4 Department of Dairy Science, University of WI, Madison, WI 53706, USA

**Keywords:** Cattle, *Mycobacteirum avium* subsp. *paratuberculosis*, Antibodies, Bulk-tank milk, ELISA

## Abstract

**Background:**

The objective of this study was to identify associations between the concentration of *Mycobacterium avium* subsp. *paratuberculosis* (MAP) antibodies in bulk milk and potential risk factors in herd management and herd characteristics, explaining high MAP antibody titers in milk. An extensive questionnaire was administered to 292 organic and conventional dairy farms from New York, Wisconsin and Oregon. Bulk milk samples were taken from each farm for MAP enzyme-linked immunosorbent assay (ELISA). A general linear model was constructed with MAP ELISA value as the outcome variable and the management factors and herd characteristics as independent variables, while at the same time controlling for the study design variables of state, herd size, and production system (organic or conventional). High bulk tank MAP ELISA value may be due to either a high prevalence of MAP in a herd with many cows contributing to the antibody titer or due to a few infected cows that produce large quantities of antibodies.

**Results:**

Results of the regression models indicated that bulk milk ELISA value was associated with season of sampling and the presence or absence of protocols for managing MAP-positive cows. The concentration of MAP antibodies in bulk milk varied seasonally with a peak in the summer and low concentrations in the winter months. When compared to farms that had never observed clinical Johne’s disease, keeping MAP-positive cows or only culling them after a period of delay was associated with an increase in optical density.

**Conclusions:**

The seasonal variation in MAP antibody titers, with a peak in the summer, may be due to a seasonal increase in MAP-bacterial load. Additionally, seasonal calving practices may contribute to seasonal fluctuations in MAP antibody titers in bulk tank milk. Keeping MAP-positive cows increases the antibody titer in bulk milk, likely due to direct antibody production in the infected cow and indirect triggering of antibody production in herdmates.

## Background

Johne’s disease, a chronic disease caused by infection with *Mycobacterium avium* subsp. *paratuberculosis* (MAP), costs the US dairy industry $200 to $250 million annually due to increased cow replacement costs and reduction in milk production [1] and also decreased fertility in high-shedding animals [2]. The control of Johne’s disease requires good herd management practices, such as preventing fecal contamination of feed and water and testing replacement cattle for MAP. Good management procedures focus on reducing transmission and the introduction of MAP into the herd [3]. Because MAP infected cows may not show clinical signs during their productive lifetime [4], it is important to test many cows in a herd to properly assess MAP infection prevalence.

A simple, quick test that provides an estimate of herd-level MAP prevalence would allow herd managers to respond by changing their management strategies, thus improving their probability of eliminating and preventing MAP infection in the long term. MAP surveillance and monitoring has been proposed as an ideal testing strategy to ensure that infection pressures are low while keeping the cost of testing low [5]. Herd-level MAP-prevalence testing often involves pooled fecal samples used for culture or PCR. However pooled sample strategies are still time-consuming because individual cows or environmental areas must be sampled [6].

The magnitude of an ELISA test result for MAP antibodies in the milk of individual cows has been reported to be related to the likelihood of an animal testing positive on a fecal culture for MAP [7]. Collins et al. [7] also reported that the level of MAP shedding, considered a measure of the stage of infection, was directly related to the ability of an individual milk ELISA test to detect an infected animal. The Parachek commercial ELISA tests have been found to have a high specificity and a sensitivity ranging from 21 to 67% on individual milk samples [8]. When used on bulk tank milk, the Pourquier ELISA test had a sensitivity of 57% [9]. Additionally, van Weering et al. [10] demonstrated that certified MAP-negative herds had a low sample/positive (S/P) ratio on bulk milk Pourquier ELISA tests and they showed that the likelihood of a herd having a MAP-infected animal increased with increasing bulk milk ELISA S/P ratio. Finally, bulk milk ELISA tests have been shown to perform similarly to serum ELISA tests at the herd level, with a sensitivity of 56 to 83%, when fecal culture is used as a reference [11]. The sensitivity can be improved by using modified protocols [8,12]. Together, these published results on bulk milk and individual cow milk provide a logical validation for the use of bulk milk ELISA corrected optical density (OD) as a continuous outcome value to scale the risk of MAP infection in the lactating herd.

Determining associations between management factors and bulk milk ELISA values will provide a simple, efficient method for farms to respond to herd MAP infections by changing their management practices or understanding risks associated with certain management practices. However, few studies have used bulk milk ELISA to identify herd-level risk factors for increased MAP prevalence, while studies relating individual animal fecal or serum test results to management practices are more common (e.g. [13,14]).

The objective of this study, therefore, is to relate bulk tank milk ELISA optical density with potential risk factors for MAP prevalence in 233 farms. Our aim was to determine if particular management practices and herd characteristics are associated with increased bulk milk ELISA values.

## Results

The median herd size was 57 for organic farms and 70 for conventional farms included in the multivariate model. Herd size ranged from 20 to 723 on the organic farms and 26 to 535 on the conventional farms. Organic farms produced an average of 14,900 lbs of milk per cow per year whereas conventional farms in the model produced an average of 20,637 lbs per cow annually. All 170 of the organic farms grazed their cattle whereas 25 conventional herds grazed and 38 conventional farms did not graze. All of the non-grazing farms allowed heifers to graze pasture.

The corrected OD, for all herds, ranged from -0.098 to 0.37 with a mean of -0.023 and a standard deviation of 0.047. The range was -0.098 to 0.17 for herds included in the multivariate model. The mean of the included herds was -0.024 and the standard deviation was 0.037. There were two farms that had a corrected OD greater than 0.17 and these were excluded from the multivariate model due to missing data.

All of the variables initially included in the multivariate model are summarized in Table 1. The stepwise order of removal from the final regression model was as follows: written plan for Johne’s disease (P = 0.95), parity (P = 0.89), calving area (P = 0.83), Jersey herd (P = 0.82), spreading manure (P = 0.75), source of drinking water (P = 0.72), open farm (P = 0.59), sine seasonality (P = 0.48), average yield (P = 0.41), heifer and cow contact on pasture (P = 0.39), Johne’s program (P = 0.43), and calf housing (P = 0.38). The variables remaining in the final multivariate model are summarized in Table 2.

**Table 1:** List of variables included in GLMSELECT procedure to produce final multivariate model

**Variable**	**Type**	**Description**
Production	Binary	Organic or Conventional
State	Nominal	NY, OR, or WI
Herd size	Ordinal	< 100, 100 to 200, or > 200
Parity	Continuous	The average lactation of all lactating cows at the time of the survey
Average yield	Continuous	Average annual milk production per cow
Calf housing	Nominal	Always housed in individual pens or areas; sometimes housed in groups or with contact to cows; always housed in groups or with contact to cows
Description of calving area	Nominal	Dedicated calving area; area shared with lactating cows; or area sometimes shared with sick cows
Heifer and cow contact on pasture	Nominal	Heifers grazing a pasture with or after the cows; before the cows; or heifers and cows do not graze the same pasture
Jersey	Binary	Mostly Jersey or not mostly Jersey herd
Johne’s procedures	Nominal	Procedures for MAP-positive cows: cull after calving or dry off; cull immediately; keep; never observed clinical Johne’s disease
Johne’s program	Binary	Participation in Johne’s program
Open farm	Binary	Some or no entering animals
Seasonality sine	Continuous	$\mathrm{Sin}\left(2\pi \frac{\mathrm{Day}}{365}\right)$ , where day is a continuous variable from 1 to 365
Seasonality cosine	Continuous	$\mathrm{Cos}\left(2\pi \frac{\mathrm{Day}}{365}\right)$ , where day is a continuous variable from 1 to 365
Source of drinking water	Nominal	Primary source of drinking water for 60 days prior to survey: well; municipal water; or surface water
Spreading manure	Binary	Manure spreading on pasture or fields that will be consumed by animals
Written plan for Johne’s disease	Binary	Written herd plan for managing Johne’s disease

**Table 2:** Summary of linear regression model

		**Multivariate model**		
**Variable**	**Description**	**β Coefficient**	**Standard error**	**P-value**
Intercept		-0.045	0.008	< .001
Production	Conventional	-0.003	0.004	0.400
	Organic	0	-	-
Herd size	< 100	0.010	0.007	0.175
	100 to 200	0.003	0.008	0.734
	> 200	0	-	-
State	NY	0.003	0.004	0.463
	OR	0.013	0.007	0.069
	WI	0	-	-
Johne’s procedures	Cull after calving or dry off	0.020	0.009	0.025
	Cull immediately	0.010	0.005	0.035
	Keep	0.018	0.007	0.019
	Never observed clinical Johne’s disease	0	-	-
Seasonality	$\mathrm{Cosine}\left(2\pi \frac{\mathrm{Day}}{365}\right)$	-0.030	0.003	< .001

Linear regression model on *Mycobacterium avium* subsp. *paratuberculosis* ELISA optical density from bulk tank milk samples of 233 farms. Multivariate model was built by backwards stepwise elimination and started with all variables listed in Table 1.

The final multivariate model residuals were approximately normal with a slight skew to the right. However, it may be expected that the PROC GLM procedure still produces valid results when the assumption of error normality is mildly violated [15].

**Figure 1:**
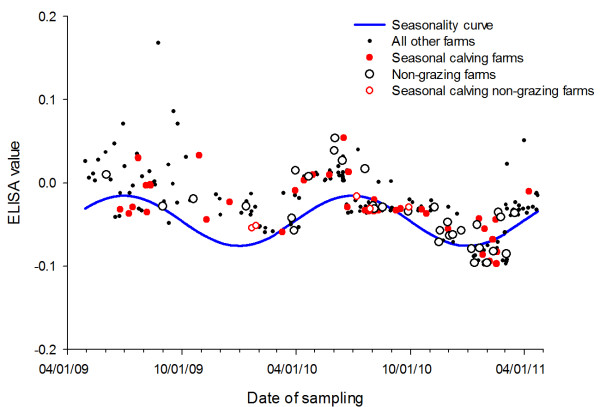
**Seasonality in ELISA optical density.** Bulk tank milk optical density of *Mycobacterium avium* subsp. *paratuberculosis* ELISA by date of sampling. Solid blue line fitted from cosine seasonality variable with all other variables set to baseline. Herds with seasonal calving practices are red dots. Open circles represent farms that do not graze their cattle. Red open circles are seasonal calving farms that do not graze. Solid dots represent all other farms included in final multivariate model.

None of the included study design variables that were forced into the models were significant in the final multivariate model (Table 2). The cosine seasonality curve was very significant (P < 0.0001). It demonstrated a peak in OD during the summer months and a trough during the winter months. The regression coefficient of -0.03 indicates the maximum drop in winter of the corrected OD and the maximum increase of +0.03 in summer. The seasonality is most evident in the farms sampled in late 2010 through 2011 (Figure 1). Seasonal calving farms and non-grazing farms were well distributed across the sampling time frame (Figure 1). There were 46 farms in the final multivariate model that indicated they used seasonal calving practices. Of these 46 farms, 42 responded that they tried to calve in the spring, 15 tried to calve in the summer, 21 in the fall and 5 in the winter. Seasonal farms did not necessarily calve over just one season, and all farms with fall or winter calving also had at least one additional calving season. The bulk milk ELISA value of seasonal calving farms differed with season of sampling, with a maximum value in the spring and continually decreasing to a minimum in the winter (Figure 1).

The procedure for managing MAP-positive cows was the only other significant variable in the multivariate model. Compared to farms that had never observed clinical Johne’s disease, farms that kept MAP-positive cows had a significant increase of 0.018 in their bulk milk OD (P = 0.019). Farms that culled MAP-positive cows also had significant increases in bulk milk ELISA value when compared to farms without a history of Johne’s disease but those that culled the cow immediately had a much smaller increase (β = 0.010, P = 0.035) than those that culled the cow after a period of delay, such as after her next calving (β = 0.020, P = 0.025). The final multivariate model explained 40% of the variation in bulk tank ELISA OD.

## Discussion

Bulk milk could have a high concentration of MAP antibodies because a dairy herd has a high prevalence of MAP infected cows or because a few infected cows produce large quantities of MAP antibodies. Variation in milk MAP antibody concentration within groups of fecal-positive and groups of fecal-negative cows has been observed due to a small number of cows producing a high level of antibodies and changes in antibody production within individual cows over time [16]. A study by van Weering et al. [10] showed that a 100-fold dilution of a positive individual milk ELISA sample may still result in a positive ELISA result, indicating that a few positive cows may impact the bulk milk ELISA result. Given the relatively small herd sizes in our study, it is clear that a few cows producing high concentrations of antibodies may influence bulk milk ELISA titers. The precise impact of a single ELISA positive cow would depend on the herd size, milk production, and the difference between the individual milk antibody concentration of a particular cow and the average antibody concentration of the rest of the herd. Therefore, some farms may have higher ELISA values than average because they have higher than average MAP infection rates or because they have some cows producing high concentrations of MAP antibodies. Careful comparison of individual cow milk ELISA tests and cow milk production level to a bulk tank test would be required to differentiate between farms that have several infected, average antibody-producing cows, and those that have a limited number of animals with high antibody titers.

In our model, the location of a farm (NY, OR, or WI) was not significantly associated with corrected OD. However, other studies have found that herds in the Midwest are more likely to be positive for Johne’s disease [14] and observe clinical signs of Johne’s disease in their herd [17]. Herd size, another study design variable, despite not being significantly associated with ELISA results in this study has previously been found to be positively associated with herd infection status, with larger herds having a greater risk [14,18]. Additionally, Wells and Wagner [14] observed a positive association between group housing for calves and the herd infection status whereas our model did not find a significant association between types of calf housing and ELISA result. Spreading manure on forage fields [13] and open water sources [19] have also been shown to be associated with a higher risk of MAP infection, but were not significantly associated with corrected OD in our model. Finally, other studies have demonstrated an increased risk of Johne’s disease with high parity and Jersey breed cows [18], although those variables did not remain in our multivariate model.

The significant seasonal effect, with MAP antibodies highest in the summer and lowest in the winter (Figure 1), represents a change in antibody secretion into bulk milk across the seasons. Seasonal calving could account for this periodic change in antibodies because milk antibodies are, on average, greatest at the beginning and end of lactation [16]. A farm that uses seasonal calving would see a herd-level increase in bulk milk antibodies, including antibodies against MAP, during or shortly after the calving season when most of their cows are just starting to lactate. This could result in high optical densities from the detection of MAP-specific and non-specific antibodies. The bulk milk ELISA value of seasonal calving farms, the majority of which indicated that they attempt to calve their cows in the spring, was greatest during the spring. This supports a days-in-milk dependent change in antibody secretion as a potential explanation for the seasonal trend in MAP antibodies in milk (Figure 1). Thus, seasonal calving could explain some of the seasonal variation but not all since only 46 of the 233 farms in the multivariate model used seasonal calving and non-seasonal calving farms still show a seasonal trend in OD (Figure 1). The seasonal variation also exists in non-grazing conventional farms (Figure 1), suggesting that the seasonal variation in MAP antibodies is not limited to grazing farms, which may have inherent seasonality in their calvings due to seasonal nutritional differences regardless of planned seasonal calving practices. Additionally, the seasonality variable remains significant (P < 0.0001) after removing all grazing farms from the final multivariate model.

Another possible explanation for the seasonal change in antibodies is a seasonal fluctuation in MAP load. It is unlikely that MAP prevalence changes seasonally on any given farm but the MAP load in the environment or in the cows could potentially change with the seasons. A previous study found a higher prevalence of MAP-positive carcasses, as determined by ileum and lymph node cultures and PCR, in the spring than in other seasons [20]. Additionally, an increase in viable MAP isolated from retail milk in the summer has been shown [21]. Humoral responses are known to occur in subclinical MAP infections, which results in activated B cells producing antibodies [22]. It has previously been suggested that MAP exposure can trigger antibody production in infection-resistant adult cattle, possibly resulting in an increased MAP ELISA titer [7]. Thus it is possible that the increase in MAP load of individual animals during the spring and summer could result in an increased humoral immune response in herdmates and therefore increase antibody levels in milk.

If bulk milk MAP ELISA is used as an indicator of MAP infection, it needs to be corrected for the seasonal changes in MAP milk antibodies. Consistently sampling during only one season may need to be recommended in order to compare ELISA results across time at one farm or among farms. Our results would need to be confirmed by similar studies to make such recommendations with more confidence.

Both culling and keeping cows known to be MAP-infected are associated with an increase in bulk milk ELISA value compared to herds without a history of clinical Johne’s disease, which suggests that farms with protocols for MAP-positive cows in place are more likely infected with MAP and possibly have a higher MAP prevalence. The larger increase in bulk milk OD associated with keeping MAP-positive cows or culling them after calving compared to the small increase in OD associated with culling MAP-positive cows immediately was not statistically significant (P = 0.25). However, it has previously been suggested that culling cows immediately is the best method for controlling MAP antibodies in bulk milk. Lu et al. [23] demonstrated the importance of culling positive animals immediately after detection in order to control MAP transmission. MAP-positive cows that are kept in the herd for any period of time could increase the bulk milk MAP antibody titer by producing MAP antibodies in their milk and by re-exposing other cows, which may then begin secreting MAP antibodies into milk. This would be particularly true for cows with a progressive course of disease, in which antibodies rise rapidly as the cow progresses from moderate to heavy fecal shedding [24].

## Conclusion

In summary, increased MAP antibody concentrations in bulk milk were associated with season of sampling and the protocols that were used on the farm for managing MAP-positive cows. The association between the season of sampling and MAP antibody concentration could be the result of seasonal variations in MAP in cows and in the environment, and could be related to seasonal calving practices. This seasonal change in MAP antibodies in bulk milk will need to be considered when using bulk milk ELISA as a MAP surveillance tool. The significance of protocols to manage MAP-positive cows pointed towards the importance of culling known MAP-positive cows immediately.

## Methods

### Study and survey

Data came from a large cross-sectional study of 292 farms conducted between March 2009 and May 2011 that focused on comparing organic and conventional dairy farms. The study has previously been described in more detail [25]. Briefly, to be included in the study, farms had at least 20 lactating cows and had been shipping milk for 2 full years prior to the study; organic farms must have been shipping certified organic milk for these 2 years. Organic herds in New York, Oregon, and Wisconsin were identified through county extension agents, personal contacts and organic certifying organizations. Conventional herds were identified from lists of licensed dairy farmers, which were obtained from the departments of agriculture in New York, Oregon and Wisconsin, and were located within 50 miles of the identified organic farms. Conventional farms were size-category matched to organic farms based on three herd-size groups: 20 to 99 cows, 100 to 199 cows, and more than 200 cows. Conventional to organic herd ratios were found to be different in each state so farms were matched accordingly: 3 organic to 1 conventional in NY, 1 organic to 1 conventional in OR, and 2 organic to 1 conventional in WI. Conventional herds were designated as non-grazing if less than 30% of the lactating cows’ dry matter intake came from pasture during the grazing season. Non-grazing farms could allow heifers to graze pasture.

A questionnaire was used on all farms to identify farm management practice and herd performance. The questionnaire has been described in detail [25] and is available online [26]. All the interviews were conducted by a single person within each state. The interviewers had been trained in administering and scoring the questionnaire in a consistent manner across states. The Institutional Review Board at Oregon State University approved the use of human subjects for the questionnaire, reference number 3995. Herds that indicated that they were seasonal calving herds were further classified by the season in which they tried to calve their cows. Seasonal calving farms were able to designate one or more seasons as a calving season on the questionnaire but the questionnaire did not require specifying a main or primary calving season. Study personnel collected bulk tank samples on the same visit as questionnaire administration. Bulk milk samples were sent to Quality Milk Production Services at Cornell University (Ithaca, NY). Samples were tested for *Salmonella spp*., *Listeria monocytogenes*, Shiga toxin producing *E. coli*, *Mycoplasma bovis*, Bovine Virus Diarrhea virus, antibodies against *Mycobacterium avium* subsp. *paratuberculosis* (MAP) and mastitis-causing bacteria. Samples were then sent to Dairy One Cooperative in Ithaca, NY for standard milk quality assays.

### Enzyme linked immunosorbent assay

Bulk tank samples were analyzed using the commercially available Parachek ELISA (product number 63308) according to the directions provided by the manufacturer (Prionics, Zurich, Switzerland). Briefly, 100 μL of each bulk milk sample was diluted with 100 μL of Green Diluent, containing *Mycobacterium phlei*, and incubated at room temperature for 30-60 minutes. Then 100 μL of each sample and 100 μL of the manufacturer-provided positive and negative controls were added to microtitre plates coated with *M. paratuberculosis*. The plates were shaken and incubated at room temperature for 30 minutes. Plates were washed 6 times with wash buffer at room temperature. Then 100 μL of conjugate reagent (Horseradish peroxidase labeled anti-bovine Ig) was added to each well. The plates were again shaken, incubated at room temperature for 30 minutes, and washed 6 times with wash buffer. Next 100 μL of enzyme substrate solution (DMSO) was added to each well and the plates were incubated and shaken at room temperature until the positive controls reached an optical density of .35 to .40 with a 620-650 nm filter. Finally, 50 μL of enzyme stopping solution (0.5 M H_2_SO_4_) was mixed into each well and the absorbance of each well was read with a 450 nm filter. The Parachek ELISA optical density result is reported as a numerical value, which is classified as positive or negative in relation to a cut-off value equal to the average negative control plus 0.10. The average optical density of the two negative controls on each plate was subtracted from the sample optical densities of the same plate [11,27] to account for inter-plate variation. This can result in the corrected optical density being less than 0 [27]. This corrected optical density value (optical density minus average negative control) was used as the continuous outcome variable for statistical analysis.

### Statistical analysis

Descriptive analyses were performed on independent and dependent variables included in the multivariate model. The key outcome variable was the corrected bulk milk ELISA optical density. Management factors and other variables of interest were identified in the dataset based on an *a-priori* rationale that they were associated with MAP antibodies in milk (Table 1). These variables of interest included aspects of herd management (production system, written plan for Johne’s disease, procedures for MAP-positive cows, participation in a Johne’s program), herd descriptors (state, herd size, average parity, average yield, Jerseys), and Johne’s disease specific risk factors (spreading manure, contact between heifers and cows on pasture, calf housing, calving area, open farm, source of drinking water). Variables to describe seasonality were developed using sine and cosine functions as previously described: $\mathrm{sine}\left(2\pi \frac{\mathrm{Day}}{365}\right)$ and $\mathrm{cosine}\left(2\pi \frac{\mathrm{Day}}{365}\right)$[28]. Stepwise backwards least-squares linear regression (PROC GLMSELECT, SAS 9.3, Inst. Inc., 2011) was used to build a multivariate model with a constant sample size of 233 farms. Fifty-nine farms were excluded from this analysis due to missing data from one or more variables evaluated for inclusion in the multivariate model. The study design variables (state, herdsize size category, production system) were forced into the model (INCLUDE option) because they determined the inclusion of herds in the study. All other variables were selected based on statistical significance at the 0.05 level. The final multivariate model was in the form:

$$\begin{array}{l} \mathrm{CORRECTED}\_\mathrm{OD}=\beta 0+\beta 1\left(\mathrm{HERDSIZE}\_<100\right)\\ \;+\beta 2(\mathrm{HERDSIZE}\_100-200)\\ \;+\beta 3(\mathrm{STATE}\_\mathrm{NY})+\beta 4(\mathrm{STATE}\_\mathrm{OR})\\ \;+\beta 5\left(\mathrm{PRODUCTION}\_\mathrm{SYSTEM}\right)\\ \;+\beta 6(\mathrm{COSINE}\_\mathrm{SEASON})\\ \;+\beta 7(\mathrm{SINE}\_\mathrm{SEASON})+\sum_{i=1}^{n}\beta_{i}X_{i}+\epsilon \end{array}$$

where OD is optical density, β_0_ is the intercept, β_1_ and β_2_ are the coefficients of the herd size variable, β_3_ and β_4_ are the coefficients of the state variable, β_5_ is the coefficient of the production system variable, β_6_ and β_7_ are the coefficients of the cosine and sine season variables respectively, β_i_ represents the coefficients of other risk factor variables X_i_ (i = 1 to n), and ϵ is a random error term assumed to be normally distributed with mean 0. This assumption was validated by examining the distribution of residuals.

## Competing interests

The authors declare that they have no competing interests.

## Authors’ contributions

CLC: performed the statistical analysis and drafted the manuscript. RMM: performed the statistical analysis and drafted the manuscript. KMCH: designed and carried out the survey. MG: designed the survey. RMR: designed and carried out the survey. PLR: designed the survey. YHS: assisted with statistical analysis and survey design, and helped draft the manuscript. All authors read and approved the final manuscript.
